# Squeezed Out: Commercial Baby Food Pouch Feeding Practices, Caregiver Perceptions, Caries Experience, and Public Health Implications

**DOI:** 10.3390/children13091174

**Published:** 2026-08-31

**Authors:** Catherine Eshaghzadeh, Suzanne Kan, Ramon Sa-a, Chi-Hong Tseng, Yan Wang, Francisco Ramos-Gomez

**Affiliations:** 1 Section of Pediatric Dentistry, School of Dentistry, University of California, Los Angeles, CA 90095, USA; s.ramon6@yahoo.com (R.S.-a.); frg@dentistry.ucla.edu (F.R.-G.); 2 Department of Medicine, School of Medicine, University of California, Los Angeles, CA 90095, USA; ctseng@mednet.ucla.edu; 3 Section of Public and Population Health, School of Dentistry, University of California, Los Angeles, CA 90095, USA; wangyan@ucla.edu

**Keywords:** early childhood caries, baby food pouches, caregiver feeding practices, infant nutrition, oral health, free sugars, public health

## Abstract

**Highlights:**

**What are the main findings?**

- Commercial baby food pouches were routinely incorporated into early childhood feeding, with two-thirds (66%) of children consuming them at least several times per week. Most children (89%) consumed pouches by direct sucking rather than by spoon or cup, and 40% sucked for five minutes or longer.
- Caregivers commonly perceived commercial baby food pouches as convenient and healthy and frequently used them during travel, as snacks, and as meal substitutes, identifying convenience as a major driver of pouch use.

**What are the implications of the main findings?**

- Frequent pouch consumption, prolonged direct sucking, and the high total sugar content of commonly consumed products may contribute to cumulative oral sugar exposure, supporting anticipatory guidance that encourages moderation and spoon or cup feeding when feasible.
- The disconnect between caregivers’ perceptions of pouch healthfulness and their feeding practices highlights opportunities for improved caregiver education, clearer nutrition labeling, and evidence-informed public health policies to promote healthier feeding practices during early childhood.

**Abstract:**

**Background/Objectives:** Early childhood caries (ECC) is the most common chronic disease of childhood worldwide. Commercial baby food pouches have become increasingly popular because of their convenience and perceived healthfulness; however, their sugar content and characteristic feeding practices have raised concerns regarding children’s oral health. This study characterized caregiver-reported commercial baby food pouch use, feeding practices, caregiver perceptions, and caries experience while considering implications for child oral health and public health policy. **Methods:** A cross-sectional study was conducted among 200 caregiver–child dyads attending the UCLA Children’s Dental Center. Eligible children were aged 0–6 years and currently consumed commercial baby food pouches. Caregivers completed a structured survey assessing pouch use, feeding practices, brand preferences, and perceptions, and children underwent oral examinations to assess caries experience using the decayed, missing, and filled teeth (dmft) index. **Results:** One-third (34%) of children consumed at least one commercial baby food pouch daily, 32% consumed pouches several times per week, and 34% consumed them less than once per week. Pouch consumption frequency differed descriptively across age groups, with approximately one-third of children aged 0–<2 years consuming pouches daily compared with approximately 20% in each older age group. Children who consumed pouches by direct, child-controlled sucking had the highest mean dmft score (5.12), followed by caregiver-controlled sucking (4.50); however, age-stratified analyses found no statistically significant differences in dmft score distributions by feeding method, frequency of pouch consumption, or time spent sucking each pouch (all *p* > 0.05). Two-thirds of caregivers (66%) reported providing pouches primarily during travel or transportation, and convenience was the most frequently cited caregiver-reported advantage. No statistically significant association was observed between caregiver education and pouch use frequency (χ^2^(20, *N* = 200) = 23.72, *p* = 0.26). The three most commonly reported pouch brands contained total sugar levels ranging from 95% to 162% of those found in a similarly sized serving of Coca-Cola, despite the pouch products reporting 0 g of added sugars on their Nutrition Facts labels. **Conclusions:** Among current pouch consumers, commercial baby food pouches were frequently incorporated into feeding practices throughout early childhood. The substantial total sugar content of commonly reported pouch products, caregiver feeding practices, and common perception as healthy products highlight opportunities for anticipatory guidance, caregiver education, clearer nutrition labeling, and public health policies that support healthier feeding practices.

## 1. Introduction

Early childhood caries (ECC) affects over 530 million children worldwide, making it one of the most prevalent childhood diseases and a significant public health concern [1]. Beyond pain and infection, ECC can adversely affect nutrition, growth, and school readiness, with disproportionate effects on vulnerable populations. Despite decades of prevention efforts, ECC remains highly prevalent across socioeconomic groups, although the burden is disproportionately borne by socially and economically disadvantaged children [1]. In the United States, according to the Centers for Disease Control and Prevention (CDC), Black and Hispanic children and those from low-income families experience approximately twice the prevalence of untreated dental caries as their peers [2].

Beyond socioeconomic disparities, the persistence of early childhood caries reflects broader changes in food systems, particularly the rapid commercialization and increased consumption of commercial baby food products. Baby food was once predominantly sold in simple jarred fruit blends with minimal additives. The introduction of squeezable pouches in 2008 marked a shift toward products formulated for convenience, enhanced palatability, and, in many cases, higher concentrations of free sugars [3]. Food pouches are commercially packaged, squeezable puree products intended for infants and young children that are typically consumed by sucking directly from an attached spout. By 2023, pouches represented nearly 40% of the global baby food market, driven by convenience, portability, and aggressive marketing [4]. These characteristics have raised concerns regarding the potential effects of commercial baby food pouches on children’s oral and overall health.

Growing evidence suggests that commercial baby food pouches may influence both oral and overall health through their nutritional composition and methods of consumption. Many commercially available pouches contain substantial amounts of sugars. Although some of these sugars are derived from processed or concentrated fruit ingredients, frequent exposure may still be relevant to oral health. They are also commonly consumed by sucking directly from the pouch, increasing the duration of sugar exposure on the teeth while limiting salivary clearance [5,6]. Frequent pouch use has also been associated with reduced exposure to textured foods, delayed oral motor skill development, selective eating behaviors, and displacement of nutrient-dense whole foods from the diet [7,8,9,10]. Together, these biological and behavioral mechanisms provide plausible pathways through which commercial baby food pouches may influence children’s oral and overall health, including the development of early childhood caries.

This exploratory, cross-sectional, mixed design study aimed to characterize commercial baby food pouch use among current pouch consumers aged 0–6 years and to examine caregiver feeding practices and perceptions relevant to children’s oral health. Specifically, we aimed to: (1) describe the frequency, duration, timing, and method of pouch consumption; (2) characterize caregiver perceptions and situations associated with pouch use; and (3) explore whether caries experience, as measured by the decayed, missing, and filled teeth (dmft) index, differed according to pouch feeding method, frequency of consumption, and time spent sucking each pouch. Given the limited existing evidence on pouch-feeding behaviors and oral health outcomes, the study was intended to be hypothesis-generating. We hypothesized that direct child-controlled sucking would be associated with greater caries experience and that convenience and perceived healthfulness would be important factors influencing caregiver use of commercial baby food pouches.

### 1.1. Nutritional Quality and Long-Term Health Implications

Baby food pouches are formulated for convenience and shelf stability but may provide lower nutritional quality than freshly prepared foods [11]. The United States Department of Agriculture (USDA) and the World Health Organization (WHO) recommend exclusive breastfeeding for approximately six months, followed by the introduction of complementary foods while limiting foods high in added sugars and sodium. Despite these recommendations, the United States has no regulations that specifically establish nutritional or promotional standards for commercial baby food pouches [5]. A 2023 evaluation of 651 commercial baby food products sold across the ten largest U.S. grocery chains found that 60% failed to meet nutritional requirements outlined by the Nutrient and Promotion Profile Model (NPPM), and none met its promotional standards [5].

Commercial processing methods used in pouch production can reduce heat-sensitive vitamins such as vitamins C and B, while their uniform texture reduces exposure to the natural fiber structure found in whole fruits and grains, which is important for digestive health and microbial diversity [7,11]. Global assessments indicate that commercially available pouched foods are often low in iron and calcium, nutrients essential for growth and neurodevelopment [7,12]. Commercial baby food pouches may also contribute to excessive sugar intake, as many products contain high levels of total sugars [5]. Although one study found no significant differences in energy intake or BMI z-scores between frequent and infrequent users, frequent pouch use was associated with greater food responsiveness, fussiness, and selective eating [9]. Evidence linking sugar restriction during the first 1000 days of life to lower risks of diabetes and hypertension in adulthood supports the Developmental Origins of Health and Disease (DOHaD) theory and underscores the long-term metabolic and oral health implications of frequent exposure to free sugars during early childhood [6,13].

### 1.2. Food Aversion and Feeding Difficulties

Frequent reliance on commercial baby food pouches during complementary feeding may contribute to feeding difficulties and food aversion. Infants typically progress from purees to textured and solid foods between 7 and 12 months of age, a critical period for developing chewing skills and oral motor coordination [14]. In contrast, consuming foods directly from pouches relies primarily on a simple forward-to-backward tongue motion and provides limited opportunity to develop the side-to-side tongue movements essential for chewing. The uniform, smooth texture also limits exposure to varied food consistencies, which may contribute to texture aversion and decreased acceptance of healthy whole foods [7,8,10,15].

This study examined commercial baby food pouch use among caregivers of children under six years of age, including patterns of use, caregiver perceptions, and feeding behaviors and their relationship with ECC. These findings are considered within a broader public health and policy context. Commercial baby food pouches represent an important intersection between convenience-driven infant feeding practices and children’s oral health, highlighting an often-overlooked opportunity for caregiver education, clinical counseling, and evidence-informed public health policy.

## 2. Materials and Methods

### 2.1. Study Population

The study was conducted at UCLA Children’s Dental Center and included children aged 0 to 6 years presenting for routine dental recall appointments. Children up to six years of age were included because commercial baby food pouches are commonly consumed beyond infancy, allowing evaluation of continued pouch use and caregiver feeding practices throughout early childhood, when ECC remains highly prevalent. Eligible participants were children classified as American Society of Anesthesiologists (ASA) Physical Status I or II (healthy children or those with mild systemic disease), having neurotypical development, no frequent consumption of sugar-sweetened beverages (≤3 times per week), and current consumption of commercial baby food pouches, as reported by the caregiver at the time of enrollment. Current pouch consumption was defined by a caregiver report that the child was currently consuming commercial baby food pouches; no minimum frequency of pouch consumption or specified look-back period was required for eligibility. Frequency of consumption was subsequently assessed as part of the survey. Thus, children who consumed pouches less than once per week remained eligible as current consumers.

### 2.2. Survey Development and Ethical Approval

A structured survey was developed based on the USDA Household Food Security Survey Module (HFSSM) and adapted to include questions specifically related to commercial baby food pouch use. The survey captured demographic information; frequency, duration, timing, and methods of pouch use (direct sucking from the pouch versus consumption by spoon or cup); preferred brands; situations in which caregivers provided pouches; caregiver perceptions of pouch healthiness; and caregiver likes and dislikes regarding pouches. Questions were designed to be culturally appropriate and easily understood by participants. The survey instrument is available from the corresponding author upon reasonable request.

The study was conducted under two UCLA Institutional Review Board approvals (BruinIRB): IRB #23-001396 (approved 4 October 2023), which covered the caregiver survey examining food pouch consumption and parental perceptions, and IRB #23-001397 (approved 28 November 2023), which covered the prospective cross-sectional observational component examining food pouch consumption and children’s dental health. All procedures adhered to ethical standards for research involving human participants.

### 2.3. Participant Recruitment and Consent

The study was conducted over a six-month period in a university-affiliated pediatric dental clinic. Dental students assisting with the study visited the clinic approximately twice per week to recruit participants. Approximately 50 patients presented to the clinic each day, of whom an estimated 20% met the inclusion criteria. During the study period, an estimated 500 caregivers were approached, and 200 agreed to participate and completed the survey (40% response rate).

Incomplete or partially completed surveys were excluded from analysis. For the open-ended questions, only participants who provided written responses were included in the qualitative analyses (*n* = 175 for “likes” and “dislikes” and *n* = 200 for “situations”), with exclusions noted below. Missing data from unanswered survey questions were handled using listwise deletion. Missingness was minimal and was considered to be missing completely at random (MCAR), resulting primarily from participants leaving individual survey items unanswered; therefore, denominators varied slightly across analyses. Given the small amount of missing data, no sensitivity analyses were performed. For analyses involving age and caries experience, 179 participants had usable linked clinical data and met the eligibility criteria applicable to the clinical component of the study. Participants without available clinical records or who did not meet the clinical eligibility criteria were excluded from these analyses.

Parents or caregivers were approached while seated in dental cubicles during their child’s recall appointment. They were invited to participate in the survey in their preferred language (English or Spanish), and informed consent was obtained in that language. Surveys were distributed during the appointments and collected after completion.

### 2.4. Oral Examination

Children received a comprehensive oral examination by dental providers within the pediatric dentistry residency clinic as part of routine clinical care. Dental caries experience was assessed using the decayed, missing, and filled teeth (dmft) index. Although providers received clinical training within the same pediatric dentistry residency program, no study-specific examiner calibration or inter-rater reliability assessment was conducted. Clinical examinations and caregiver surveys were conducted separately; caregiver surveys were collected by research volunteers, and dental providers did not have access to participants’ survey responses. Formal blinding was not implemented, as caregivers could independently discuss their child’s dietary and feeding practices with the dental provider as part of routine nutritional counseling during the clinical visit.

### 2.5. Statistical Analysis

Frequency distributions were generated for sociodemographic variables and pouch consumption habits. Associations between categorical variables, including caregiver education level and frequency of pouch use, were analyzed using the chi-square test of independence. The dmft was summarized using the median and interquartile range (IQR). Differences in dmft score distributions by feeding method, frequency of pouch consumption, and time spent sucking each pouch were assessed using Kruskal–Wallis tests stratified by age category. All statistical tests were two-tailed, with statistical significance set at *p* < 0.05.

### 2.6. Qualitative Analysis

Several open-ended questions were included in the survey to explore caregiver perspectives on baby food pouch use, including “What do you like most about baby food pouches?”, “What do you like least about baby food pouches?”, and “In what situations do you provide your child with a food pouch?” Responses were analyzed using an inductive qualitative content analysis approach. Two investigators independently reviewed and manually coded all written responses into recurring thematic categories; no specialized qualitative analysis software was used. Individual responses could be assigned to more than one category when multiple ideas were expressed. Responses that were off-topic, unclear, or uninterpretable were excluded from analysis (13 for “likes,” 22 for “dislikes,” and 9 for “situations”). Descriptive frequencies (*n*, %) were calculated to indicate the proportion of respondents mentioning each theme. Because participants could contribute to more than one theme, percentages exceed 100%.

### 2.7. Sugar Content Comparison of Commonly Reported Pouch Brands

To provide descriptive context regarding the sugar content of pouch products most commonly reported by caregivers, nutrition information was examined for the three most frequently reported brands: GoGo SqueeZ (Materne North America, New York, NY, USA), Gerber Baby (Gerber Products Company, Arlington, VA, USA), and Kirkland Signature (Costco Wholesale Corporation, Issaquah, WA, USA). Total and added sugar content was obtained from U.S. Nutrition Facts information published by the manufacturers for pouch products evaluated during the study period and was recorded as grams per pouch. For GoGo SqueeZ, the Apple Apple product depicted in the survey instrument contained 13 g of total sugars per 90 g pouch. Gerber Baby pouch varieties evaluated during the study period contained 10–17 g of total sugars per 99 g pouch, and both Kirkland Signature pouch varieties evaluated contained 10 g of total sugars per 90 g pouch. All evaluated pouch products reported 0 g of added sugars per pouch.

For comparison, Coca-Cola (The Coca-Cola Company, Atlanta, GA, USA) was used as a reference, with a total sugar content of 10.6 g per 100 mL. For each pouch product, total sugar content per pouch was compared with the total sugar content of an approximately equivalent quantity of Coca-Cola using the following calculation: (total sugar content of the pouch ÷ total sugar content of an equivalent quantity of Coca-Cola) × 100. When multiple pouch varieties within a brand were evaluated, the observed range of total sugar content was reported. These comparisons were intended to provide descriptive context regarding the total sugar content of commonly reported pouch products and were not used as exposure variables in the statistical analyses.

## 3. Results

### 3.1. Participant Characteristics

A total of 200 caregiver–child dyads were included in the analysis. Most caregivers were married (65%), identified as Latino/Hispanic (64%), and spoke English (61%) or Spanish (32%) at home. Educational attainment varied, with 61% reporting at least some college education, and most caregivers (60%) reported no difficulty meeting basic household expenses (Table 1). Children were predominantly 2–4 years of age (*n* = 96, 54%), followed by those aged 5–6 (*n* = 71, 40%), and those under 2 years (*n* = 12, 6.7%).

### 3.2. Commercial Baby Food Pouch Consumption Patterns

Patterns of commercial baby food pouch use are summarized in Table 2. Commercial baby food pouch use was reported across children of all ages, ethnicities, and socioeconomic strata represented in the study. Most children (60%) used pouches for less than five minutes per feeding occasion, but a substantial proportion (32%) sucked from pouches for 5–30 min, with nearly 8% extending beyond 30 min. The majority (69%) sucked directly from the pouch while holding it themselves, whereas 20% sucked directly from pouches held by a caregiver, and only 11% consumed pouch contents using a spoon or cup. Pouch use was most frequent in the mid-afternoon (45%) and at lunchtime (31%). These patterns of pouch use by consumption method, duration, and time of day were generally consistent across age groups, whereas consumption frequency varied by age. Overall, one-third (34%) of children in our study consumed at least one pouch per day, 32% consumed pouches several times per week, and the remaining one-third (34%) consumed pouches less than once per week. Among children aged 0–<2 years, approximately one-third reported daily pouch consumption, compared with approximately 20% in each of the older age groups. In contrast, consuming pouches less than once per week was the most common frequency of consumption for children aged 5–6 years (38%) and 2–4 years (29%). Caregivers also commonly reported providing pouches as snacks or meal substitutes (Table 3).

### 3.3. Association Between Caregiver Education and Food Pouch Consumption

**Figure 1:**
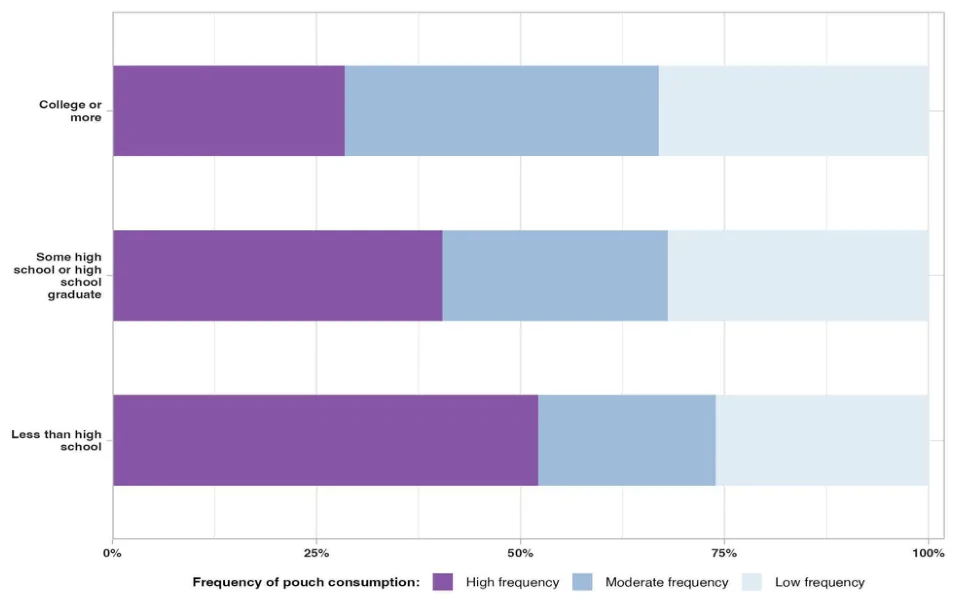
Caregiver Education level and Frequency of Food Pouch Consumption.

No statistically significant association was observed between caregiver education level and frequency of food pouch consumption (χ^2^(20, *N* = 200) = 23.72, *p* = 0.26). Across all frequency groups, caregivers who were college graduates represented the largest educational category. More than half (52%) of caregivers with less than a high school education reported high frequency of food pouch use, in contrast with 40% of high school graduates, and one-third (28%) of college educated parents (Figure 1).

### 3.4. Method of Pouch Consumption and Caries Experience

**Figure 2:**
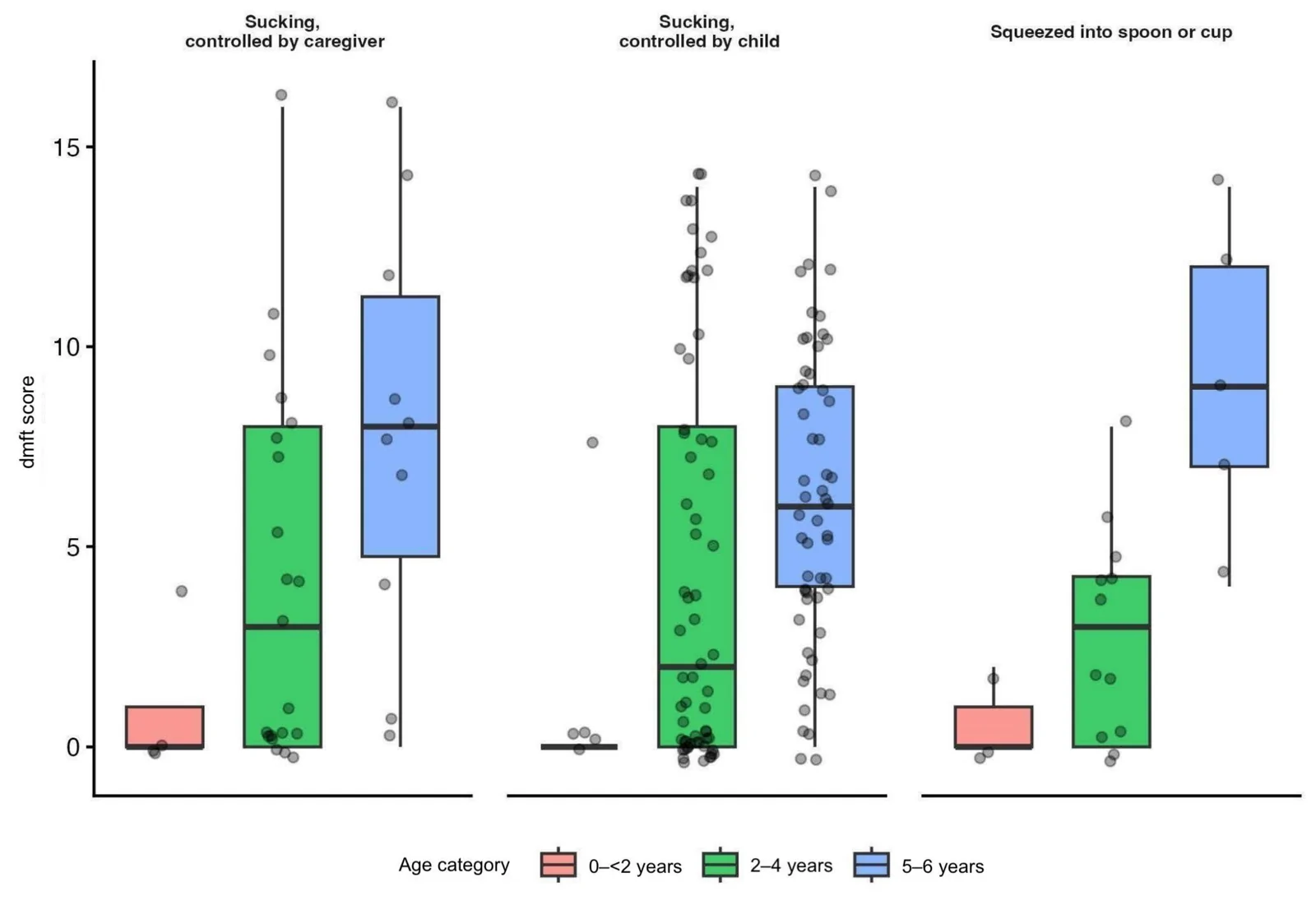
Method of Pouch Consumption in Relation to dmft scores, by age category. Individual points represent observed dmft scores; darker points reflect overlapping observations.

Analysis compared dmft scores by feeding method, frequency of consumption, and time spent sucking each food pouch to examine associations with caries experience. Among the 200 caregiver–child dyads who completed the survey, 179 were included in analyses involving age and dmft. Overall, the median dmft score was 4 (IQR = 0–8). Children who sucked directly from pouches they held and controlled themselves had the highest mean dmft score (5.12, SD = 4.55), followed by those fed through caregiver-controlled sucking (mean dmft = 4.5, SD = 5.07). However, differences in dmft distributions across feeding methods were not statistically significant (Kruskal–Wallis *H* = 0.73, *p* = 0.39). As age may confound the relationship between pouch-use characteristics and caries, analyses were stratified by age category. Age-stratified distributions of dmft scores by feeding method are shown in Figure 2. Median dmft scores differed descriptively across age groups, from 0 (IQR: 0–0.3) among children aged <2 years, to 8 (IQR: 0–8) among children aged 2–4 years, and 5 (IQR: 4–9) among children aged 5–6 years. Within age-strata, Kruskal–Wallis tests found no statistically significant differences in dmft score distributions by feeding method, frequency of consumption, or time spent sucking each pouch (all *p* > 0.05).

### 3.5. Situations in Which Caregivers Provide Food Pouches

All caregiver respondents (*n* = 200) answered the question, “In what situations do you provide your child with a food pouch?” After excluding nine off-topic or uninterpretable comments, 191 valid responses were analyzed (Table 3). The majority of caregivers (*n* = 126, 66%) reported using food pouches during travel, vacations, trips, or while in a transportation vehicle. Other common situations included providing pouches at a specific or scheduled time (*n* = 30, 16%), as a substitute for a regular meal when one could not be provided (*n* = 28, 15%), or as a snack (*n* = 18, 9%). A smaller subset reported using pouches for behavior control to soothe or occupy a child who was impatient, bored, or upset (*n* = 14, 7%) or as positive reinforcement when a child was behaving well (*n* = 14, 7%). Percentages were calculated using the 191 valid responses as the denominator and may exceed 100% because caregivers could report more than one situation.

### 3.6. Caregiver-Reported Likes of Baby Food Pouches

Of the 200 caregiver respondents, 175 provided open-ended responses to the question, “What do you like most about baby food pouches?” After excluding 13 off-topic or uninterpretable comments, 162 valid responses were analyzed (Table 4). The most frequently cited theme was convenience, reflecting portability and practicality across settings (*n* = 63, 39%), followed by ease of use, reflecting simplicity of feeding or handling (*n* = 52, 32%), and the perception that pouches are healthy (*n* = 42, 26%). A smaller proportion of caregivers specifically emphasized the speed or time-saving nature of pouch use (*n* = 12, 7%) or reported that their child enjoys the taste (*n* = 8, 5%). Percentages exceeded 100% because respondents could contribute to more than one thematic category.

### 3.7. Caregiver-Reported Dislikes of Baby Food Pouches

A total of 175 caregiver respondents answered the question, “What do you like least about baby food pouches?” After excluding 22 off-topic or uninterpretable comments, 153 valid responses were analyzed (Table 5). The most common theme was no concerns reported or overall satisfaction, mentioned by 62 caregivers (41%). Among those who reported dislikes, the most frequently cited issue was high sugar content (*n* = 37, 24%), followed by packaging and practicality (e.g., plastic waste, messiness, insufficient food per package, preservation; *n* = 20, 13%) and nutrition- and health-related concerns (e.g., limited nutrient density, inadequate satiety, preservatives, or perceived overreliance; *n* = 16, 10%). Other recurring themes included cost (*n* = 9, 6%), taste and variety (*n* = 4, 3%), developmental and feeding behavior concerns (*n* = 4, 3%), and safety and quality concerns (*n* = 3, 2%). Percentages were calculated using the 153 valid responses as the denominator and may exceed 100% because participants could mention more than one theme.

## 4. Discussion

The results characterize behavioral and broader structural factors related to commercial baby food pouch use and patterns of caries experience among children who consume pouches. Our findings are consistent with previous studies reporting that baby food pouches have transitioned from an occasional convenience product to a routine component of infant feeding. Rowan et al. [16] found that caregivers valued pouches primarily for convenience, portability, and reduced mess, while concerns about nutrition were often secondary. Similarly, convenience was the most frequently cited advantage reported by caregivers in our study, suggesting that practical caregiving demands may outweigh nutritional considerations in feeding decisions.

The predominance of direct sucking, particularly child-controlled sucking, creates prolonged and repeated sugar exposure within the oral cavity, amplifying caries risk through continuous demineralization periods and reduced salivary clearance. These findings align with the broader understanding that feeding style and exposure duration are important determinants of ECC, not solely the quantity of sugar consumed.

Although differences in dmft score distributions across feeding methods did not reach statistical significance, children who sucked directly from pouches demonstrated the highest mean dmft scores. This pattern is biologically plausible and consistent with the established pathogenesis of early childhood caries. Direct sucking from a pouch encourages repeated, prolonged exposure of the teeth to fermentable carbohydrates while limiting salivary clearance, particularly when children self-feed over extended periods. In contrast, spoon or cup feeding typically results in shorter oral exposure times and allows for more efficient clearance of food residues. Frequent sipping or grazing behaviors repeatedly lower plaque pH below the critical threshold for enamel demineralization, as described by the Stephan curve, extending the duration of acid attacks and increasing cumulative cariogenic exposure [17,18].

Although pouch consumption occurred across all education levels, caregivers with lower educational attainment appeared more likely to report high-frequency pouch use. Parents working multiple jobs or with limited support may rely on convenient, ready-to-use foods to meet daily caregiving demands. Additionally, caregivers may perceive fruit pouches as healthy or equivalent to whole foods because of their fruit-based ingredients and health-oriented marketing. The appeal of pouches—convenience, portability, and ease of use—mirrors many characteristics associated with fast food, reinforcing their role as a practical solution in modern feeding practices [16]. Although statistical differences were not observed, the directional trend suggests that convenience-driven reliance on pouches may be more common among caregivers facing greater time or resource constraints.

Consumption patterns observed in this study suggest that commercial baby food pouches have become a routine component of young children’s diets rather than an occasional convenience food. Approximately two-thirds (66%) of children consumed pouches at least several times per week, including one-third (34%) who consumed them daily and 13.5% who consumed two or more pouches per day. Given the substantial sugar content of many commercial baby food pouches, this pattern of frequent exposure may contribute to repeated acid attacks and cumulative cariogenic challenges during a critical period of oral development, particularly when combined with prolonged, child-controlled sucking behaviors.

Understanding these patterns is especially important in the context of caregiver behavior. Caregivers most commonly reported providing pouches during travel, vacations, or transportation, followed by at a specific or scheduled time and as a substitute for a regular meal when one could not be provided. These findings suggest that convenience is a major driver of frequent pouch use, with pouches functioning as portable, low-mess feeding options in situations where traditional meals may be difficult to provide. Although our survey did not directly assess daycare or school use, similar demands for convenience and behavioral management may also influence reliance on commercial baby food pouches in institutional settings. These patterns suggest that frequent pouch use is shaped not only by caregiver preferences but also by broader structural factors, including time constraints, mobility, and modern caregiving demands.

Open-ended caregiver responses further illustrate these dynamics. The qualities most frequently valued were convenience, ease of use, and speed. When asked what they liked least, some caregivers identified high sugar content or nutritional concerns, whereas a substantial proportion reported no concerns at all. These findings suggest that even when caregivers recognize nutritional shortcomings, perceived convenience may outweigh health considerations. As long as commercial pouch products provide an appealing combination of portability, ease of use, and behavioral convenience, families may continue to rely on them despite concerns regarding sugar content and nutritional quality.

Among caregivers in our study, the three most commonly reported pouch brands were GoGo SqueeZ (*n* = 108, 54.0%), Gerber Baby (*n* = 81, 40.5%), and Kirkland Signature/Costco Brand (*n* = 58, 29.0%). Based on manufacturer-published U.S. Nutrition Facts information evaluated during the study period, these products contained total sugar levels ranging from 95% to 162% of the total sugar content of an approximately equivalent quantity of Coca-Cola (10.6 g/100 mL). GoGo SqueeZ Apple Apple, the brand’s original applesauce flavor, contained 13 g of total sugars per 90 g pouch (137%), Kirkland Signature pouch varieties contained 10 g per 90 g pouch (105%), and the evaluated Gerber Baby varieties contained 10–17 g per 99 g pouch (95–162%). Notably, the evaluated pouch products reported 0 g of added sugars on their Nutrition Facts labels, whereas the sugars in Coca-Cola are added sugars. This distinction highlights that products marketed as containing no added sugars may nevertheless contain substantial amounts of total sugars. From an oral health perspective, the absence of added sugars does not necessarily indicate low cariogenic potential, as caries risk is influenced by the availability of fermentable carbohydrates as well as the frequency and duration of exposure and oral clearance.

Taken together, these findings raise concerns about how health-oriented marketing may shape caregiver perceptions of commercial baby food pouches. Claims such as “natural,” “organic,” or “no added sugar” may be interpreted as indicating low cariogenic risk, even when products contain substantial amounts of total sugars. The distinction between added and total sugars may therefore be particularly important in caregiver interpretation of product healthfulness. Clearer nutrition labeling and evidence-based marketing standards may help caregivers distinguish pouch products from whole foods and make more informed feeding decisions.

This study has several limitations. Survey responses relied on caregiver self-report, which may be influenced by recall or social desirability bias, potentially leading to underreporting of unhealthy feeding practices. Although oral examinations were conducted by providers trained within the same pediatric dentistry residency program, no study-specific examiner calibration or inter-rater reliability assessment was performed, which may have introduced variability in dmft assessment. While the study excluded children who consumed sugar-sweetened beverages more than three times per week, this criterion may not have fully accounted for variation in other cariogenic dietary exposures. Other dietary habits and oral hygiene practices may also affect caries risk, resulting in potential residual confounding. Because eligibility was restricted to children who currently consumed commercial baby food pouches, the study did not include a non-pouch-consuming comparison group and therefore cannot determine whether pouch consumption itself is associated with caries experience. Analyses of caries experience were limited to comparisons among pouch consumers according to feeding method, frequency of pouch consumption, and time spent sucking each pouch. Complete age and dmft data were available for a subset of participants, and the resulting reduced sample, together with relatively small and uneven age-stratified group sizes, limited statistical power to detect potentially meaningful differences in dmft across pouch-use characteristics. The study population was drawn from a single dental center and may not represent the broader population. Additionally, the cross-sectional design limits causal inference, and variations in caregiver interpretation of survey questions, as well as the exclusion of caregivers unable to complete the survey in English or Spanish may further affect the generalizability of the findings. Statistical power was also limited for detecting differences across caregiver education levels. Caries experience is strongly associated with age, as older children have had more time to develop caries. We therefore stratified analyses by age to account for potential age-related differences, but the uneven strata and small sample size, particularly in the 0 < 2 year age group, limited the precision of age-specific estimates and the ability to detect meaningful associations. Larger, more balanced studies are needed to determine whether the observed differences represent true associations and to better characterize the relationships between caregiver feeding practices and oral health outcomes.

### 4.1. Policy and Public Health Implications

Addressing commercial baby food pouch consumption requires coordinated action across clinical, community, industry, and policy levels. Pediatricians, pediatric dentists, dietitians, and other healthcare providers should counsel families on appropriate pouch use, encourage spoon or cup feeding when feasible, promote moderation, and support the gradual introduction of textured whole foods. Manufacturers and policymakers should improve transparency by clearly labeling sugar content, limiting potentially misleading nutrition claims, and ensuring marketing practices accurately reflect evidence-based infant and young child feeding recommendations. Because caregivers in this study most commonly reported using pouches during travel and other convenience-driven situations, educational efforts should emphasize realistic, healthier alternatives that are practical for busy families rather than simply discouraging pouch use. Integrating oral health into existing nutrition programs, including WIC and SNAP education initiatives, could further strengthen caregiver awareness of healthy feeding practices and support healthier feeding behaviors during early childhood. Community health workers and caregiver education programs also have an important role in helping families identify affordable, practical alternatives that promote both children’s oral health and overall development.

### 4.2. Future Research

Future research should include non-pouch-consuming comparison groups and longitudinal designs to clarify whether commercial baby food pouch use and specific pouch feeding practices are associated with ECC incidence over time. Standardized dietary assessment tools are needed to capture pouch consumption accurately in national surveys. Studies exploring the impact of pouch feeding on the oral microbiome could reveal novel biological pathways. Finally, cross-cultural analyses of pouch marketing and adoption would provide insight into global patterns and inform international policy approaches.

## 5. Conclusions

In this study, commercial baby food pouch use was common across all demographic groups, with two-thirds of children consuming pouches at least several times per week and most children consuming them by sucking directly from the pouch. Caregivers most often valued pouches for their convenience and ease of use and frequently perceived them as healthy, despite the high sugar content of the most commonly reported brands.

Commercial baby food pouches may represent an underrecognized source of substantial sugar exposure during a critical stage of child development. Their convenience and health-oriented marketing may contribute to caregiver underestimation of potential risks to both oral and overall health.

While these products are used across all socioeconomic groups, they may be relied upon more frequently by families facing time, financial, or caregiving constraints. In these settings, frequent pouch use may unintentionally increase prolonged sugar exposure and contribute to oral health disparities already experienced by underserved children.

Addressing this challenge requires coordinated efforts that extend beyond the dental office, including caregiver education, anticipatory guidance from healthcare providers, clearer nutrition labeling, responsible marketing practices, and public health policies that support healthier and more accessible feeding options. Recognizing pouch feeding practices as a potentially modifiable source of frequent or prolonged oral sugar exposure represents an opportunity to support healthier feeding practices and reduce preventable oral health risks during early childhood.

## Figures and Tables

**Table 1:** Caregiver Demographics and Household Information.

Caregiver Variables		*n*	Percent (%)
Marital Status			
	Married	130	65
	Member of unmarried couple	17	8
	Single/Divorced/Widowed/Other	53	26
Race/Ethnicity			
	African American or Black	12	6
	Asian	19	10
	Caucasian/White/European American	25	12
	Latino/Latina/Latin American/Hispanic	128	64
	Multiethnic or Mixed	14	7
	Native American/Pacific Islander/Hawaiian	2	1
Highest Level of Education			
	Elementary (Grades 1–8 or less)	25	12
	High School or GED (Grades 9–12)	52	26
	Some College or Technical School (1–3 years)	69	34
	College Graduate (4+ years)	54	27
Language Spoken at Home			
	English	121	61
	Spanish	62	31
	Other Languages	17	8
Economic Stability (Ability to pay for food, housing, medical care)			
	No difficulty making ends meet	119	60
	Sometimes Difficult	60	30
	Difficult to make ends meet	21	10

**Table 2:** Food Pouch Consumption Habits.

Food Pouch Consumption Habits	*n*	Percent
Frequency		
High Frequency	68	34
>3 times a day	6	3
2–3 times a day	21	10.5
Once a day	41	20.5
Moderate Frequency	64	32
Almost every day	13	6.5
A couple days a week	51	25.5
Low Frequency	68	34
Less than once a week	68	34
Time spent for each occasion the child sucks from the food pouch		
Less than 5 min	120	60
5–30 min	64	32
30–1 h	9	4.5
More than an hour	7	3.5
Method of food pouch consumption		
Sucking directly from the food pouch held and controlled by caregiver	40	20
Sucking directly from the food pouch held and controlled by child	138	69
With contents squeezed onto a spoon or cup	22	11
Time of day		
Breakfast	26	11
Mid-morning	66	27
Lunch	74	31
Mid-afternoon	109	45
Dinner	19	8

**Table 3:** Situations in Which Caregivers Provide Food Pouches.

Situations Caregiver Provides Child with Food Pouch	*n*	Percent (%)
During travel/vacations/trips/in any transportation vehicle	126	66
At a specific/scheduled time	30	16
As a substitute for a regular meal when it cannot be provided	28	15
Snack	18	9
Behavior control (for soothing purposes, times when a child is impatient, bored or angry)	14	7
When they are behaving well (positive reinforcement)	14	7

Of the 200 participants who provided a response to this question, 9 comments were excluded because they were off-topic or not interpretable. Percentages are calculated from the 191 valid responses. Percentages may exceed 100% because respondents could contribute to more than one thematic category.

**Table 4:** Caregiver-Reported Likes of Baby Food Pouches.

What Caregivers Like the Most About Baby Food Pouches	*n*	Percent (%)
Convenience	63	39
Easy	52	32
Fast	12	7
Healthy	42	26
Taste	8	5

Of the 175 participants who provided a response to this question, 13 comments were excluded because they were off-topic or not interpretable. Percentages are calculated from the 162 valid responses. Percentages may exceed 100% because respondents could contribute to more than one thematic category. Convenience reflected portability and practicality across settings; ease of use reflected simplicity of feeding or handling; and fast reflected time-saving or rapid feeding/preparation.

**Table 5:** Caregiver-Reported Dislikes of Baby Food Pouches.

What Caregivers Like the Least About Baby Food Pouches	*n*	Percent (%)
Nothing/I like everything about them	62	41
High sugar content (addictive)	37	24
Nutrition (not enough nutrition, not satiating)	16	10
Cost (Expensive)	9	6
Packaging (Plastic, messy, not enough food per package, preservation)	20	13
Taste (Flavors)	4	3
Safety & Quality (Mold/Lead)	3	2
Feeding Behavior (Not chewing, not solid, eating too quickly)	4	3

Of the 175 participants who provided a response to this question, 22 comments were excluded because they were off-topic or not interpretable. Percentages are calculated from the 153 valid responses. Percentages may exceed 100% because respondents could contribute to more than one thematic category.

## Data Availability

The data presented in this study are available from the corresponding author upon reasonable request. The data are not publicly available because they contain information that could compromise participant privacy.

## References

[1] World Health Organization Ending Childhood Dental Caries: WHO Implementation Manual. https://www.who.int/publications-detail-redirect/ending-childhood-dental-caries-who-implementation-manual.

[2] Edelstein B.L. (2025). Chronic Disease Management of Early Childhood Dental Caries. Prev. Chronic Dis..

[3] de Souza M.S., Vaz J.D.S., Martins-Silva T., Bomfim R.A., Cascaes A.M. (2021). Ultra-Processed Foods and Early Childhood Caries in 0–3-Year-Olds Enrolled at Primary Healthcare Centers in Southern Brazil. Public Health Nutr..

[4] Baby Food Packaging Market Demand & Trends 2025–2035. Future Market Insights. https://www.futuremarketinsights.com/reports/baby-food-packaging-market.

[5] Coyle D.H., Shahid M., Parkins K., Hu M., Padovan M., Dunford E.K. (2024). An Evaluation of the Nutritional and Promotional Profile of Commercial Foods for Infants and Toddlers in the United States. Nutrients.

[6] Gračner T., Boone C., Gertler P.J. (2024). Exposure to sugar rationing in the first 1000 days of life protected against chronic disease. Science.

[7] Koletzko B., Bührer C., Ensenauer R., Jochum F., Kalhoff H., Lawrenz B., Körner A., Mihatsch W., Rudloff S., Zimmer K.-P. (2019). Complementary foods in baby food pouches: Position statement from the Nutrition Commission of the German Society for Pediatrics and Adolescent Medicine (DGKJ, e.V.). Mol. Cell. Pediatr..

[8] Coulthard H., Harris G., Emmett P. (2009). Delayed introduction of lumpy foods to children during the complementary feeding period affects child’s eating behavior at 7 years of age. Matern. Child. Nutr..

[9] Cox A.M., Taylor R.W., Haszard J.J., Beck K.L., von Hurst P.R., Conlon C.A., Te Morenga L., Daniels L., McArthur J., Paul R. (2024). Baby food pouches and Baby-Led Weaning: Associations with energy intake, eating behavior, and infant weight status. Appetite.

[10] Harris G., Coulthard H. (2016). Early Eating Behaviours and Food Acceptance Revisited: Breastfeeding and Introduction of Complementary Foods as Predictive of Food Acceptance. Curr. Obes. Rep..

[11] Reddy M.B., Love M. (1999). The impact of food processing on the nutritional quality of vitamins and minerals. Adv. Exp. Med. Biol..

[12] World Health Organization Systematic Review of Unhealthy Foods and Beverages. https://cdn.who.int/media/docs/default-source/nutrition-and-food-safety/complementary-feeding/cf-guidelines/systematic-review-unhealthy-foods-and-beverages.pdf.

[13] Lacagnina S. (2019). The Developmental Origins of Health and Disease (DOHaD). Am. J. Lifestyle Med..

[14] Perez-Escamilla R., Segura-Perez S., Lott M. (2017). Feeding Guidelines for Infants and Young Toddlers: A Responsive Parenting Approach. Nutr. Today.

[15] Northstone K., Emmett P., Nethersole F., ALSPAC Study Team (2001). The effect of age of introduction to lumpy solids on foods eaten and reported feeding difficulties at 6 and 15 months. J. Hum. Nutr. Diet..

[16] Rowan M., Mirosa M., Heath A.-L.M., Katiforis I., Taylor R.W., Skeaff S.A. (2022). A Qualitative Study of Parental Perceptions of Baby Food Pouches: A Netnographic Analysis. Nutrients.

[17] Stephan R.M. (1940). Changes in hydrogen-ion concentration on tooth surfaces and in carious lesions. J. Am. Dent. Assoc..

[18] Fejerskov O., Nyvad B., Kidd E.A.M. (2015). Dental Caries: The Disease and Its Clinical Management.

